# RETRACTED: Yen et al. Brevilin A Ameliorates Imiquimod-Induced Psoriasis-like Dermatitis and Reduces Th17 Differentiation in Psoriasis Patients. *J. Pers. Med.* 2022, *12*, 1888

**DOI:** 10.3390/jpm15020072

**Published:** 2025-02-18

**Authors:** Ling-Jung Yen, Chung-Yang Yen, Chia-Ling Li, En-Chih Liao, Kai-Chun Wang, Meng-Chieh Shih, Hung-Sen Huang, Ying-Chin Chen, Ling-Ying Lu, Sheng-Jie Yu

**Affiliations:** 1 Division of Allergy, Immunology, and Rheumatology, Department of Internal Medicine, Kaohsiung Veterans General Hospital, Kaohsiung 813, Taiwan; 2 Department of Nursing, Meiho University, Pingtung 912009, Taiwan; 3 Department of Dermatology, Taichung Veterans General Hospital, Taichung 407, Taiwan; 4 School of Medicine, National Yang Ming Chiao Tung University, Taipei 112, Taiwan; 5 Children’s Medical Center, Taichung Veterans General Hospital, Taichung 407, Taiwan; 6 Institute of Biomedical Sciences, MacKay Medical College, New Taipei City 25245, Taiwan; 7 Department of Medicine, MacKay Medical College, New Taipei City 25245, Taiwan; 8 The Doctoral Program of Clinical and Experimental Medicine, National Sun Yat-Sen University, Kaohsiung 804, Taiwan; 9 Department of Medical Education and Research, Kaohsiung Veterans General Hospital, Kaohsiung 813, Taiwan; 10 Institute of Biomedical Sciences, National Sun Yat-Sen University, Kaohsiung 804, Taiwan; 11 Department of Medical Research, Taichung Veterans General Hospital, Taichung 407, Taiwan

The journal retracts the article “Brevilin A Ameliorates Imiquimod-Induced Psoriasis-like Dermatitis and Reduces Th17 Differentiation in Psoriasis Patients” [1], cited above.

Following publication, the authors identified an issue related to the reported mouse strain used in the study and informed the Editorial Office. The original manuscript stated that C57BL/6 mice were used in the in vivo experiments, whereas the correct strain should be BALB/c mice. To address this, the authors submitted supporting documents to verify the experiments were conducted using another mouse strain.

Adhering to our complaints procedure, an investigation was conducted that confirmed that the evidence provided by the authors was not sufficient to rectify the concerns regarding the use of animal subjects in the experiments. As a result, the Editorial Board have agreed to retract this article [1], as per MDPI’s retraction policy (https://www.mdpi.com/ethics#_bookmark30, accessed on 15 October 2024).

This retraction was approved by the Editor-in-Chief of the *Journal of Personalized Medicine*.

The authors did not agree to this retraction.
